# Integrating traditional machine learning with qPCR validation to identify solid drug targets in pancreatic cancer: a 5-gene signature study

**DOI:** 10.3389/fphar.2024.1539120

**Published:** 2025-01-09

**Authors:** Xiaoyan Wang, Pengcheng Yu, Wei Jia, Bingbing Wan, Zhougui Ling, Yangyang Tang

**Affiliations:** ^1^ Key Laboratory of Systems Biomedicine (Ministry of Education), Shanghai Center for Systems Biomedicine, Shanghai Jiao Tong University, Shanghai, China; ^2^ Department of General Surgery, The Fourth Affiliated Hospital of Guangxi Medical University, Liuzhou, China; ^3^ Department of Pulmonary and Critical Care Medicine, The Fourth Affiliated Hospital of Guangxi Medical University, Liuzhou, China

**Keywords:** pancreatic cancer, biomarkers, peripheral blood, drug targets, machine learning

## Abstract

**Background:**

Pancreatic cancer remains one of the deadliest malignancies, largely due to its late diagnosis and lack of effective therapeutic targets.

**Materials and methods:**

Using traditional machine learning methods, including random-effects meta-analysis and forward-search optimization, we developed a robust signature validated across 14 publicly available datasets, achieving a summary AUC of 0.99 in training datasets and 0.89 in external validation datasets. To further validate its clinical relevance, we analyzed 55 peripheral blood samples from pancreatic cancer patients and healthy controls using qPCR.

**Results:**

This study identifies and validates a novel five-gene transcriptomic signature (LAMC2, TSPAN1, MYO1E, MYOF, and SULF1) as both diagnostic biomarkers and potential drug targets for pancreatic cancer. The differential expression of these genes was confirmed, demonstrating their utility in distinguishing cancer from normal conditions with an AUC of 0.83. These findings establish the five-gene signature as a promising tool for both early, non-invasive diagnostics and the identification of actionable drug targets.

**Conclusion:**

A five-gene signature is established robustly and has utility in diagnostics and therapeutic targeting. These findings lay a foundation for developing diagnostic tests and targeted therapies, potentially offering a pathway toward improved outcomes in pancreatic cancer management.

## 1 Introduction

Pancreatic cancer persists as one of the most lethal malignancies worldwide, with a devastating 5-year survival rate of less than 9% (Zhao and Liu, 2020). This poor prognosis is largely attributed to late-stage diagnosis and limited therapeutic options, underscoring the urgent need for both early detection methods and novel therapeutic targets (Mizrahi et al., 2020). Despite significant advances in cancer research, the identification of reliable biomarkers and effective drug targets for pancreatic cancer has proven challenging, primarily due to its complex molecular heterogeneity and the rapid development of drug resistance (Costello et al., 2012; Mottini et al., 2024). Traditional approaches to biomarker and drug target discovery have relied heavily on tissue-based analyses, which, while informative, present significant limitations including invasiveness and the challenge of repeated sampling (Batis et al., 2021). Recent advances in molecular biology and bioinformatics have opened new avenues for discovery, particularly through the analysis of large-scale genomic datasets (Clark and Lillard, 2024; Shi et al., 2024). However, the translation of computationally identified targets to clinically viable options remain a significant challenge, with many promising candidates failing in later validation stages.

The emergence of liquid biopsy approaches, particularly blood-based testing, has revolutionized cancer diagnostics by offering a minimally invasive method for disease monitoring (Fu et al., 2024; Shegekar et al., 2023; Chen et al., 2024). This approach is particularly relevant for pancreatic cancer, where traditional tissue acquisition is often complicated by anatomical location and associated risks. However, the development of reliable blood-based markers requires robust validation across multiple platforms and patient cohorts. Machine learning approaches have demonstrated remarkable potential in identifying complex molecular signatures from large-scale datasets (Glaab et al., 2021). Traditional machine learning methods, particularly those focusing on feature selection and meta-analysis, offer several advantages over newer deep learning approaches, including interpretability and the ability to handle heterogeneous data sources. These methods are particularly valuable in biomarker discovery, where understanding the biological relevance of selected features is crucial for downstream drug development. Previous studies have attempted to identify gene signatures for pancreatic cancer, but most have suffered from several limitations: (1) small sample sizes leading to poor generalizability, (2) lack of independent validation in different patient cohorts, (3) limited validation in easily accessible biological samples such as blood, and (4) insufficient evaluation of the identified genes as potential therapeutic targets (Strijker et al., 2019). Additionally, many studies have focused solely on diagnostic potential without considering the therapeutic implications of their findings (Kirkegård et al., 2017).

Our study addresses these limitations through a comprehensive approach that combines traditional machine learning methods with experimental validation. By analyzing 14 independent pancreatic cancer datasets comprising 845 samples, we aimed to identify a robust gene signature that could serve both diagnostic and therapeutic purposes. Our approach employs random-effects meta-analysis and forward-search optimization to ensure the selected genes demonstrate consistent differential expression across multiple cohorts, reducing the risk of dataset-specific artifacts. Importantly, we extend beyond computational prediction by validating our findings in peripheral blood samples from 55 subjects, addressing a critical gap in the field–the need for minimally invasive diagnostic tools. This validation step not only confirms the clinical utility of our signature but also demonstrates the potential of these genes as therapeutic targets, as their expression is detectable in peripheral blood.

Our study’s unique strength lies in its dual focus on both diagnostic and therapeutic applications. The identified five-gene signature (LAMC2, TSPAN1, MYO1E, MYOF, and SULF1) represents not just a diagnostic tool but also a set of potential drug targets. Each of these genes has been implicated in various aspects of cancer biology, suggesting their potential as therapeutic targets. LAMC2, for instance, plays a crucial role in cancer cell invasion and metastasis, while SULF1 is involved in multiple signaling pathways critical for tumor progression. The present study aims to validate this signature through a rigorous, multi-step approach that combines computational analysis with experimental validation. Our findings could significantly impact both the early detection of pancreatic cancer and the development of targeted therapies, potentially addressing two of the most critical challenges in pancreatic cancer management.

## 2 Materials and methods

### 2.1 Data sources and computational analysis

We systematically searched and identified 14 prospective pancreatic cancer studies from the GEO database (http://www.ncbi.nlm.nih.gov/geo/). The datasets included GSE15471, GSE16515, GSE23397, GSE28735, GSE32676, GSE39751, GSE55643, GSE56560, GSE60979, GSE62165, GSE62452, GSE63111, GSE71989, GSE91035, and GSE15932, encompassing a total of 845 subjects (Table 1). We performed a random split of these datasets, allocating 50% to a training and validation set, with two prospect independent test sets (GSE91035, GSE15932) reserved for external validation (Table 1). Also, we collect in-house validation dataset. All transcriptomic data underwent normalization using the GC-Robust Multi-array Average (gcRMA) algorithm, followed by log2 transformation of gene expression values prior to analysis. To minimize between-trial variance, we employed the DerSimonian-Laird random-effects method to combine gene expression effect sizes using Hedges’ g effect size calculations. Through this analysis, we identified significant genes based on effect size (ES > 2) and Fisher’s method false discovery rate (FDR <0.01).

**TABLE 1:** Demographic of the datasets in training and external validation.

GEO ID	Sample size	Sample type	Description
GSE15471	36	tumor tissue	Training
GSE23397	21	tumor tissue	Training
GSE39751	24	tumor tissue	Training
GSE56560	35	tumor tissue	Training
GSE62165	131	tumor tissue	Training
GSE63111	35	tumor tissue	Training
GSE71989	22	tumor tissue	Training
GSE16515	52	tumor tissue	Validation
GSE28735	90	tumor tissue	Validation
GSE32676	32	tumor tissue	Validation
GSE55643	53	tumor tissue	Validation
GSE60979	93	tumor tissue	Validation
GSE62452	130	tumor tissue	Validation
GSE91035	59	tumor tissue	Validation
GSE15932	32	peripheral blood	Validation

### 2.2 Patient recruitment and sample collection

Between January 2023 and December 2023, we recruited 55 participants for this study. The study group comprised 30 patients with histologically confirmed pancreatic ductal adenocarcinoma and 25 healthy controls (Supplementary Table S1). Inclusion criteria for pancreatic cancer patients included: age between 18–75 years, histologically confirmed pancreatic ductal adenocarcinoma, no prior cancer treatment, and adequate organ function. Exclusion criteria encompassed: presence of other malignancies, severe organ dysfunction, active infection, autoimmune diseases, or a history of chronic or heavy alcohol consumption. Healthy controls were age- and gender-matched individuals with no history of cancer or chronic diseases. All blood samples were collected under standardized conditions to minimize variability. Specifically, blood was drawn in the early morning between 7:00 and 9:00 a.m. after an overnight fast of at least 8 h. This study was approved by the ethical committee of The Fourth Affiliated Hospital of Guangxi Medical University (Approval No. KY2023309), and all participants signed informed consent forms prior to enrollment.

### 2.3 Blood sample processing and RNA extraction

Peripheral blood samples (5 mL) were collected in EDTA tubes from all participants following standard venipuncture procedures. Blood samples were processed within 2 h of collection. Total RNA was isolated using the TRIzol LS reagent (Invitrogen) following the manufacturer’s protocol. RNA quality and quantity were assessed using a NanoDrop spectrophotometer and Agilent 2100 Bioanalyzer. Only samples with RNA integrity number (RIN) > 7 were used for subsequent analysis.

### 2.4 Quantitative PCR analysis

First-strand cDNA was synthesized from 1 μg of total RNA using the SuperScript III First-Strand Synthesis System (Invitrogen). Quantitative PCR was performed using SYBR Green Master Mix (Applied Biosystems) on an ABI 7900HT Real-Time PCR System. Each reaction was performed in triplicate. GAPDH served as the internal control for normalization. The primer sequences for the five target genes (LAMC2, TSPAN1, MYO1E, MYOF, and SULF1) were designed and validated for specificity and efficiency (Supplementary Table S2). PCR conditions included initial denaturation at 95°C for 10 min, followed by 40 cycles of 95°C for 15 s and 60°C for 1 min. Relative gene expression was calculated using the 2^-ΔΔCt^ method.

### 2.5 Gene expression data and statistics analyses

All transcriptomic data were normalized using the GC-Robust Multi-array Average (gcRMA) algorithm (Wu and Irizarry, 2004). A log2 transformation was applied to all gene expression before analysis. To underestimates the between-trial variance, we used the DerSimonian-Laird random-effects combine gene expression effect sizes via Hedges’g effect size (Henmi and Copas, 2010; Enzmann, 2015). Moreover, based on gene effect size (ES > 2), and Fisher’s method false discovery rate (FDR <0.01), we identified a subset of genes as the pancreatic cancer signature.

### 2.6 Creation of pancreatic cancer signature

To construct the pancreatic cancer signature, we employed the forward search algorithm available in the MetaIntegrator R package, which facilitates the selection of an optimal gene set for diagnostic purposes. Forward search is a stepwise feature selection method that incrementally builds a gene set by maximizing diagnostic performance, measured by the weighted area under the receiver operating characteristic curve (AUC). As a starting point, we ran a forward search using the MetaIntegrator R package to identify the parsimonious gene set best suited for diagnostic ability (Haynes et al., 2017). The process starts by identifying the single gene with the highest individual discriminative ability, determined by its weighted AUC across datasets. This gene serves as the foundation of the model. Subsequently, the algorithm iteratively evaluates all remaining genes and adds the one that provides the greatest incremental improvement to the overall weighted AUC when combined with the previously selected genes. This stepwise addition of genes continues until no further gene can significantly improve the weighted AUC beyond a predefined threshold. At each iteration, the pancreatic cancer signature was calculated using the following formula: Pancreatic Cancer Signature = Mean (expression of upregulated genes)−Mean (expression of downregulated genes). This algorithm ensures that the final gene set is both minimal and robust, avoiding overfitting and maintaining diagnostic accuracy across diverse datasets.

## 3 Results

### 3.1 The five diagnostic biomarkers of pancreatic cancer in six training datasets

**FIGURE 1:**
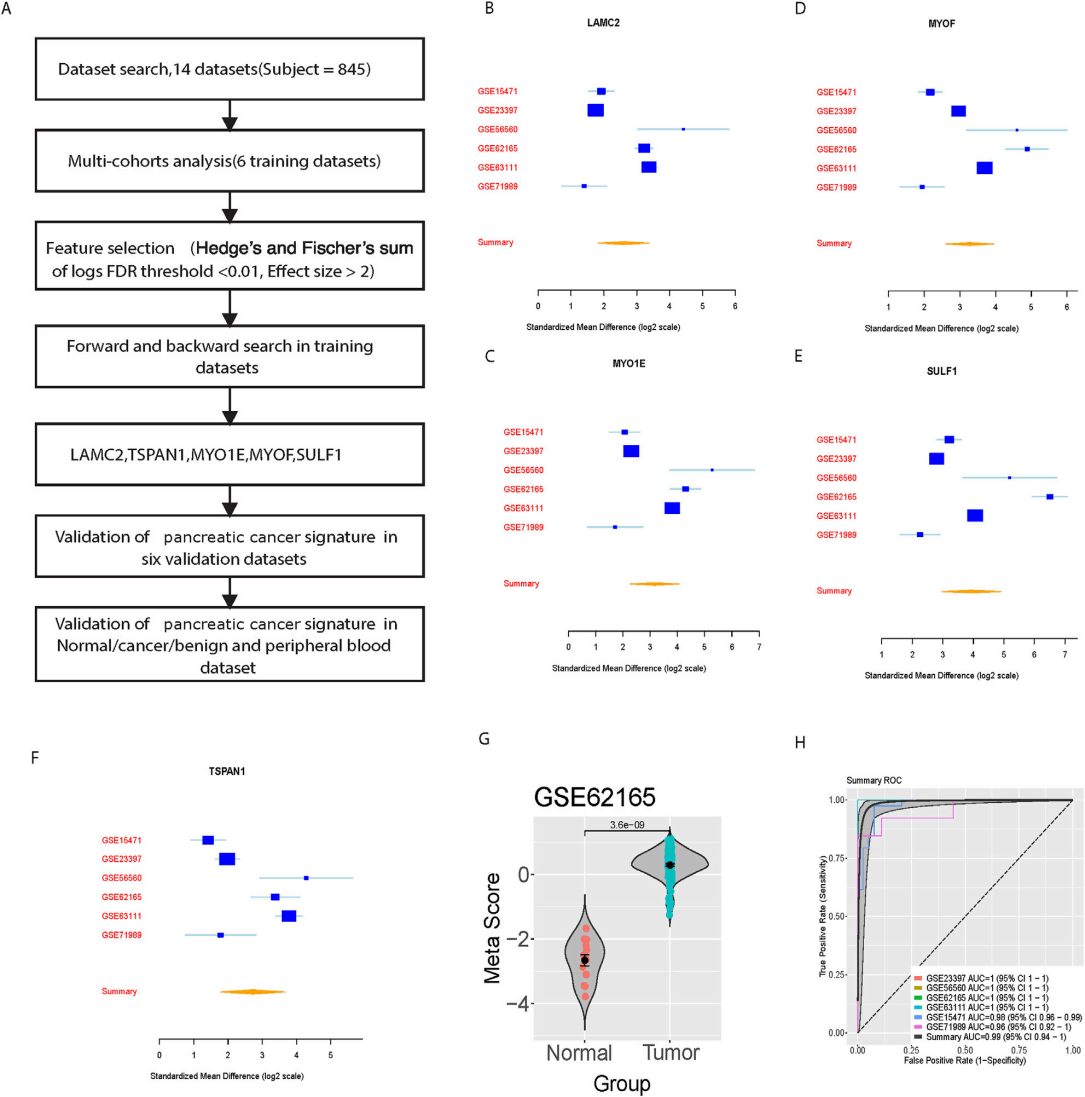
Identification and training validation of the five-gene signature for pancreatic cancer diagnosis. **(A)** Workflow diagram showing the systematic approach for signature identification. **(B–F)** Forest plots showing standardized mean differences (log2 scale) for individual genes (LAMC2, TSPAN1, MYO1E, MYOF, SULF1) across six training datasets. **(G)** Violin plot showing meta-score distribution between normal and tumor samples in GSE62165. **(H)** Summary ROC curves showing diagnostic performance across training datasets with individual and combined AUCs.

We achieved a systematic search for data on transcriptome-wide expression between normal and pancreatic cancer tissue. According to the previously described method (ES > 2, FDR <0.01), 23 genes were significantly upregulated, while 48 genes were significantly downregulated (Figure 1A). After forward search and backward search, we identified a set of five differentially expressed genes (LAMC2, TSPAN1, MYO1E, MYOF, SULF1) in Cancer/Normal that was optimized for diagnostic ability (Figures 1B–F). The pancreatic cancer signature was calculated for each sample by Mean (upregulated genes) – Mean (downregulated genes) (Figure 1G). The pancreatic cancer signature (five diagnostic biomarkers) distinguished cancer from normal subjects with a summary area under the curve (AUC) = 0.99 [95% CI 0.94–1] in six training datasets (Figure 1H).

### 3.2 Validation of the five diagnostic biomarkers in six external dataset of pancreatic cancer patients

**FIGURE 2:**
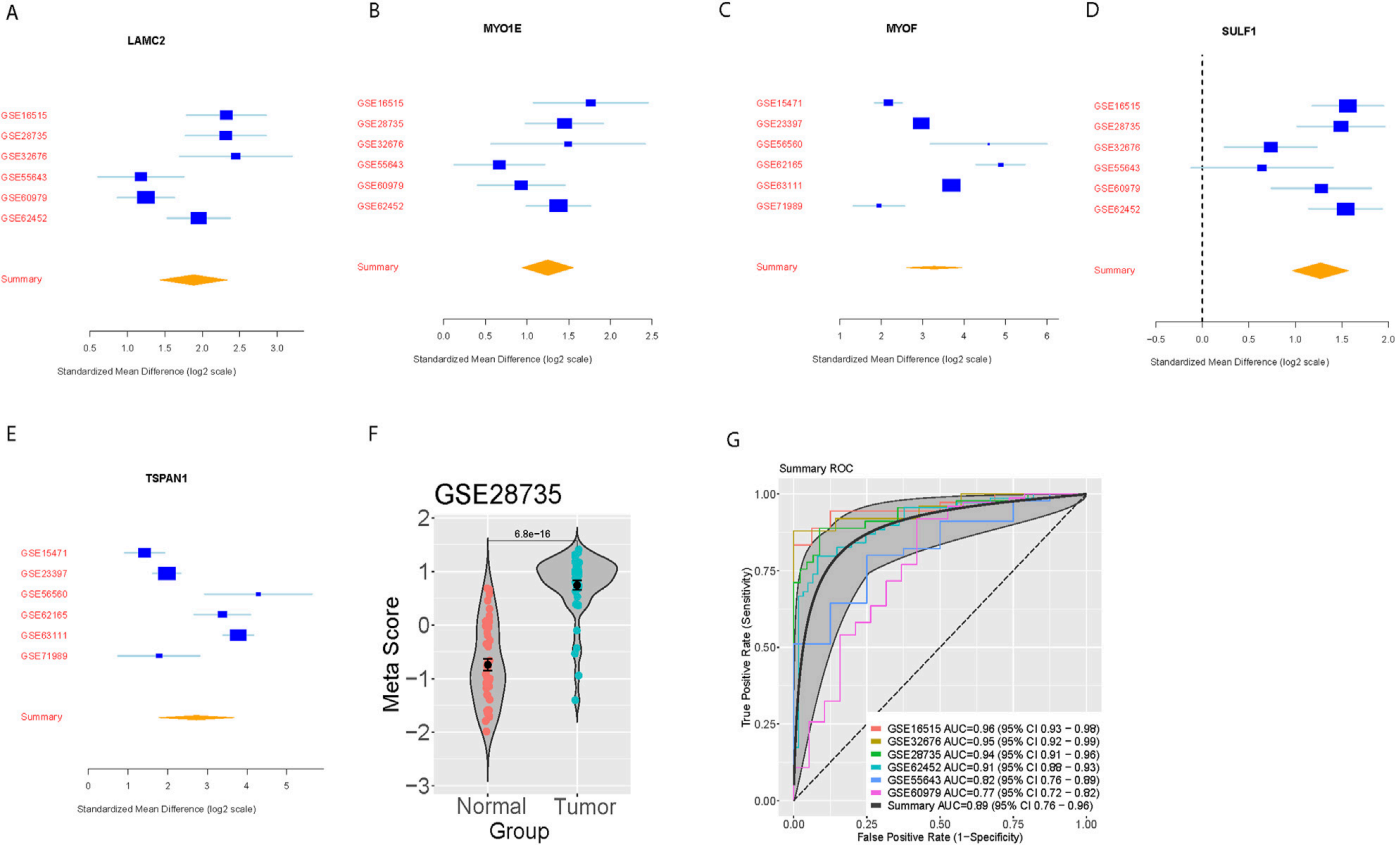
External validation of the five-gene signature in independent datasets. **(A–E)** Forest plots showing standardized mean differences (log2 scale) for each signature gene across six validation datasets. **(F)** Violin plot showing meta-score distribution between normal and tumor samples in GSE28735. **(G)** Summary ROC curves demonstrating diagnostic performance across validation datasets with individual and combined AUCs.

We further verified the pancreatic cancer signature in the six-validation set. For each dataset, we computed the effect size and meta-score by the previously described method (Figures 2A–F). A summary area under the curve (AUC) = 0.89 [95% CI 0.76–0.96] differential cancer from normal subjects by the pancreatic cancer signature in six training datasets (Figure 2G).

### 3.3 Validation of the five diagnostic gene biomarkers in cancer/benign and peripheral blood dataset

**FIGURE 3:**
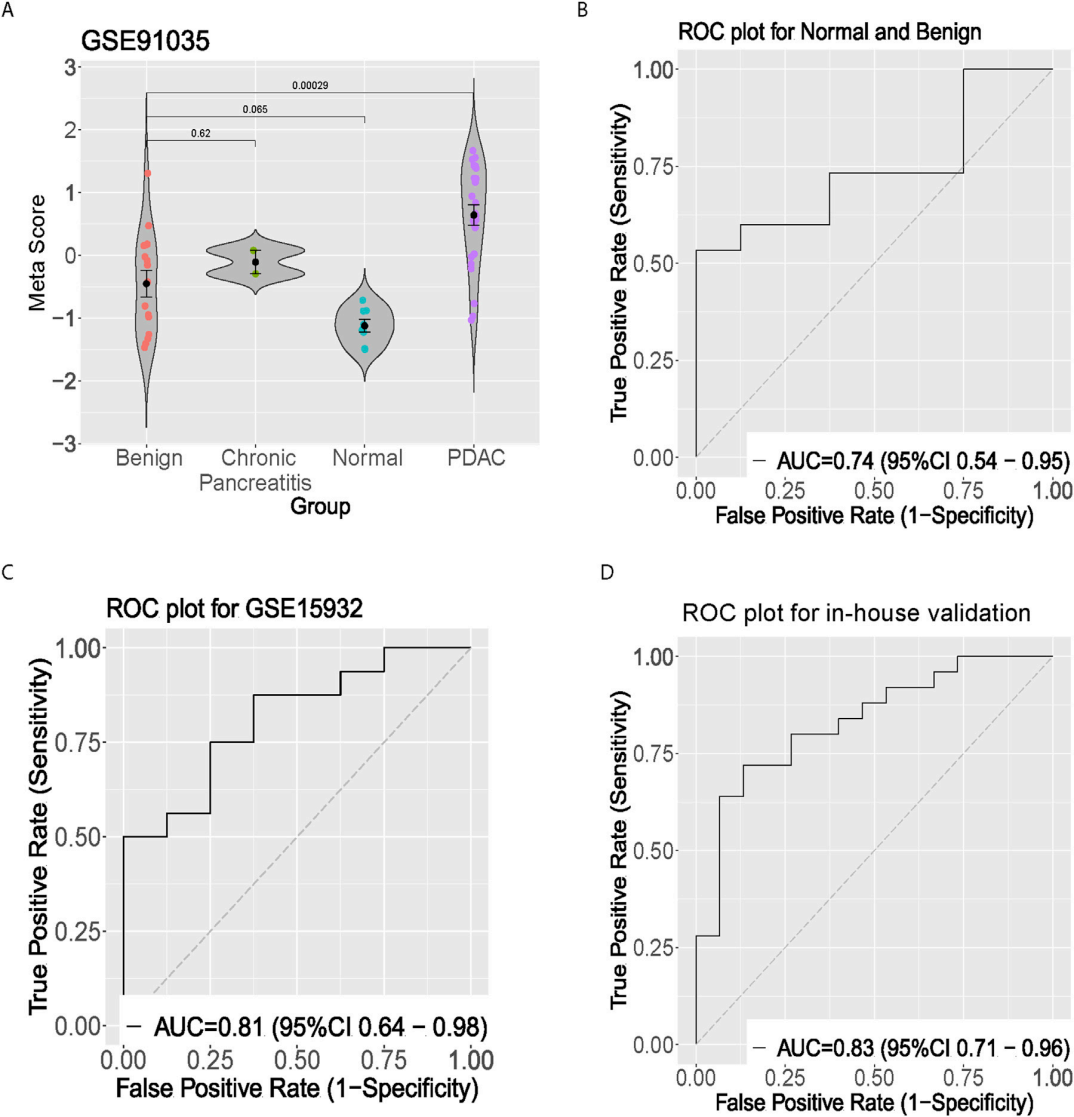
Clinical validation of the five-gene signature in peripheral blood samples. **(A)** Violin plot showing meta-score distribution across benign, chronic pancreatitis, normal, and PDAC groups in GSE91035 dataset. **(B)** ROC curve showing discrimination between normal and benign samples. **(C)** ROC curve for GSE15932 peripheral blood validation. **(D)** ROC curve from in-house validation cohort demonstrating diagnostic performance in distinguishing pancreatic cancer from healthy controls.

To further evaluate the diagnostic power, a two prospects independent test set was performed. In the first prospect dataset (GSE91035), we calculated the meta-score in the normal, benign, and pancreatic cancer groups, respectively (Figure 3A). Interestingly, the meta score was proportional to the severity of pancreatic cancer. There were significant differences between the Benign and pancreatic cancer groups (*p* < 0.01). Since there is no significant difference between the normal and the benign group, a separation trend can be seen (*p* = 0.065) (Figure 3B). The pancreatic cancer signature distinguished normal from benign subjects with AUC = 0.74 [95% CI 0.54–0.95] and distinguished normal from cancer in in-house validation cohort with AUC = 0.83 [95% CI 0.71–0.96] (Figures 3C, D).

## 4 Discussion

Our study presents a comprehensive analysis establishing a novel five-gene signature for pancreatic cancer diagnosis and therapeutic targeting, with validation across multiple datasets and platforms. The robust performance of this signature, particularly in peripheral blood samples, represents a significant advance in non-invasive pancreatic cancer diagnostics and potential therapeutic development. The exceptional performance of our signature in both training (AUC = 0.99) and validation datasets (AUC = 0.89) demonstrates its robust diagnostic capability across diverse patient populations. Notably, the successful validation in peripheral blood samples (AUC = 0.83) suggests potential clinical utility as a non-invasive diagnostic tool. This is particularly significant for pancreatic cancer, where early detection remains challenging due to anatomical location and non-specific symptoms.

Each gene in our signature plays crucial biological roles in cancer progression. LAMC2, encoding a laminin subunit, is a critical component of the laminin-5 complex involved in regulating cell adhesion, migration, and tumor microenvironment remodeling. Elevated LAMC2 expression promotes pancreatic cancer invasion and metastasis by enhancing AKT-dependent NHE1 activity (Wang et al., 2020). TSPAN1, a tetraspanin family member, modulates cell surface protein trafficking and signaling, contributing to cancer cell proliferation and chemoresistance through pathways such as EGFR and integrin-mediated signaling (Garcia-Mayea et al., 2022). MYO1E and MYOF, involved in actin-based intracellular trafficking and vesicle transport, respectively, play key roles in cancer cell membrane dynamics and migration. Dysregulation of these genes has been linked to enhanced metastatic potential and altered intracellular signaling in pancreatic cancer (Chung et al., 2019). SULF1, an extracellular heparan sulfate endosulfatase, regulates key growth factor pathways such as FGF2 and VEGF, impacting angiogenesis and tumor progression (Nagamine et al., 2012). These genes collectively influence the tumor microenvironment, facilitating processes such as immune evasion, extracellular matrix degradation, and angiogenesis.

In addition to their biological relevance, we compared the diagnostic performance of our five-gene signature to CA19-9, the most used biomarker for pancreatic cancer. While CA19-9 demonstrates high specificity for pancreatic cancer, its sensitivity is limited, particularly in early-stage disease (Azizian et al., 2020). In contrast, our gene signature achieved comparable diagnostic accuracy with AUC = 0.83 in peripheral blood samples, offering the advantage of a non-invasive, molecular-based approach. Furthermore, CA19-9 is influenced by non-cancerous conditions such as cholestasis, which may lead to false-positive results. The integration of our signature with CA19-9 could potentially enhance diagnostic precision by combining molecular and traditional markers. Future studies should explore the combined utility of these biomarkers.

The validation of our signature in peripheral blood samples addresses a critical need in pancreatic cancer management. Current diagnostic methods often rely on invasive procedures or imaging techniques with limited sensitivity for early-stage disease. Our blood-based approach could facilitate regular screening and monitoring, potentially enabling earlier detection and improved treatment outcomes. The ability to detect these markers in blood also suggests their potential utility in monitoring treatment response and disease progression.

Our study’s dual focus on diagnostics and therapeutic targeting represents a novel approach in biomarker development. While most previous studies have focused solely on diagnostic applications, our identification of genes with known roles in cancer biology opens new avenues for targeted therapy development. The consistent differential expression of these genes across multiple datasets and their detection in peripheral blood suggests their fundamental importance in pancreatic cancer pathogenesis. The use of machine learning approaches, particularly random-effects meta-analysis and forward-search optimization enabled robust signature development while minimizing dataset-specific biases. This methodological approach could serve as a template for similar biomarker discovery efforts in other cancers. The successful validation across multiple independent datasets and platforms strengthens the generalizability of our findings.

However, several limitations warrant discussion. First, while our blood-based validation included 55 subjects, larger prospective studies are needed to fully establish clinical utility. Second, the relationship between tissue and blood expression levels requires further investigation to optimize blood-based testing protocols. Third, functional studies are needed to fully understand the therapeutic potential of targeting these genes. Future directions should include prospective validation in larger, diverse patient cohorts, particularly focusing on early-stage disease detection. Investigation of the mechanistic roles of these genes in pancreatic cancer progression could inform therapeutic development strategies. Finally, longitudinal studies are needed to explore the utility of the gene signature in monitoring disease progression and treatment response. Additionally, studies examining the signature’s utility in monitoring treatment response and predicting prognosis would expand its clinical applications.

## 5 Conclusion

Our study establishes a robust five-gene signature with demonstrated utility in both diagnostics and therapeutic targeting. The successful validation in peripheral blood samples represents a significant step toward non-invasive pancreatic cancer detection. Our findings provide a foundation for future development of both diagnostic tests and targeted therapies, potentially improving outcomes in this devastating disease.

## Data Availability

The datasets presented in this study can be found in online repositories. The names of the repository/repositories and accession number(s) can be found in the article/Supplementary Material.
